# Outcome Assessment after Reconstruction of Tumor-Related Mandibular Defects Using Free Vascularized Fibular Flap—A Clinical Study

**DOI:** 10.3390/healthcare11020193

**Published:** 2023-01-09

**Authors:** Zahid Qayyum, Zafar Ali Khan, Afsheen Maqsood, Namdeo Prabhu, Mohammed Saad Alqarni, Alzarea K. Bader, Rakhi Issrani, Maria Shakoor Abbasi, Naseer Ahmed, Mohammed Ghazi Sghaireen, Artak Heboyan

**Affiliations:** 1 Department of Oral & Maxillofacial Surgery, Khyber Girls Medical College, Hayatabad Medical Complex, Peshawar 23301, Pakistan; 2 Department of Oral & Maxillofacial Surgery and Diagnostic Sciences, College of Dentistry, Jouf University, Sakaka 72388, Saudi Arabia; 3 Department of Oral Pathology, Bahria University Dental College, Karachi 75530, Pakistan; 4 Department of Prosthetic Dental Sciences, College of Dentistry, Jouf University, Sakaka 72388, Saudi Arabia; 5 Department of Preventive Dentistry, College of Dentistry, Jouf University, Sakaka 72388, Saudi Arabia; 6 Department of Prosthodontics, Altamash Institute of Dental Medicine, Karachi 75500, Pakistan; 7 Department of Prosthodontics, Faculty of Stomatology, Yerevan State Medical University after Mkhitar Heratsi, Str. Koryun 2, Yerevan 0025, Armenia

**Keywords:** autogenous graft, fibula flap reconstruction, oral health impact

## Abstract

The objective of this study was to analyze the outcomes of the free vascularized fibular flap in the reconstruction of mandibular defects, and to assess the oral health impact profile of these patients before surgery and after oral rehabilitation. Patients requiring reconstruction of defects greater than 6 cm were selected for this study. The defect size and type, the size of the required skin paddle, the need for second flaps, the intraoperative complications, and the type of closure were documented. Patients were evaluated postoperatively for function, aesthetics, and donor- or reconstruction-site complications. The validated oral health impact profile (OHIP-14) questionnaires were filled before and after surgery and after dental rehabilitation. This study included 11 cases of squamous-cell carcinomas, 2 cases of malignant nerve sheath tumors, and 1 case each of malignant melanoma, ameloblastoma, giant-cell tumor, osteosarcoma, and chondrosarcoma. The analysis revealed a significant association (*p* = 0.030) of gender with free vascular flap complications, while no significant association (*p* > 0.05) was found when donor- and recipient- site complications, as well as the type of resection (Brown’s classification), were compared with free vascular flaps. Moreover, the total OHIP-14 scores for patients before surgery, after surgery, and after dental rehabilitation were 12.03 ± 1.34, 10.66 ± 1.41, and 08.33 ± 0.62, respectively. The oral health-related quality of life was markedly improved after the reconstruction of the mandibular defects with free vascularized fibular flap and dental rehabilitation. The overall success rate of fibular flap in our study was 72.2%, which is lower than that reported in the literature. This may be attributed to the fact that almost all of our cases included large segmental defects that extended across the midline of the mandible.

## 1. Introduction

Several reconstructive challenges arise after tissue loss ensuing surgical oncological resection in the maxillofacial region. The resultant complex soft- and hard-tissue defect mandates restoration with a flap that contains vascularized bone muscles and skin for optimal esthetics and function [1,2,3]. The main objective of the reconstruction of maxillofacial defects is immediate repair in one single-stage operation with well-vascularized tissue and a low complication rate [3,4].

In the past, the reconstruction of maxillofacial defects had remained compromised before the introduction of vascularized tissue flap. It included the placement of reconstruction plates joining the defect segments, which were augmented with non-vascularized bone grafts, but they would often be placed without any bone graft. The thin and mobile oral mucosa pulled down against the narrow edge of the plate due to the weight of the remaining underlying muscles or the bulky pedicle flaps used for soft tissue reconstruction, which usually caused plate exposure, especially after radiotherapy, in such patients [5,6].

The free fibula flap (FFF) is currently the cornerstone in the head–neck reconstruction armamentarium since its first development in 1975 by Tylor et al. [7] and subsequent introduction in the reconstruction of the oromandibular region by Hidalgo D.A. [8]. Today, FFF is considered as the gold standard in mandibular reconstruction due to its ability to be harvested with multiple skin paddles allowing paramount form and function establishment, high success, and a low complication rate at both recipient and donor sites [2,9].

The FFF has an advantageous dual endosteal and periosteal blood supply, making it a reliable reconstruction option in multiple complicated osteotomies to adapt the mandibular contour [10]. It can provide up to 25 cm of bone length, which is sufficient for lengthy mandibular defect reconstructions with thick cortical bone, ideal for interosseous fixative plates, screws, and dental implant osteointegration. This renders it the first reconstruction choice for the head and neck surgeon [11].

The majority of the studies conducted in the past were retrospective. These studies may have overestimated the survival rate because of selection bias, or complications might have been underreported in some studies due to a lack of proper documentation. Moreover, so far only a few retrospective studies have evaluated the quality of life of these patients, but not at all three stages (preoperatively, postoperatively, and after dental rehabilitation). The authors found no statistically significant changes in the quality of life after dental rehabilitation except for the social aspect [12,13].

In the present study, our research question was whether mandibular reconstruction utilizing the vascularized fibular flap has a better outcome and can significantly improve the oral health-related quality of life of these patients before surgery, after surgery, and after dental rehabilitation. The purpose of this study was to analyze the outcome of mandibular reconstruction utilizing the vascularized fibular flap in patients, and to assess their oral health-related quality of life before surgery, after surgery, and after dental rehabilitation.

## 2. Materials and Methods

### 2.1. Study Setting and Ethical Approval

This prospective study was conducted at the Hayatabad medical complex, Peshawar, Pakistan, on patients requiring resection of their lower jaw. The study was approved by the Ethics Committee (protocol number: 561/HEC/B&PSC). The ethical aspects of the Declaration of Helsinki were followed.

### 2.2. Study Protocol

The operation plan, surgical risks, treatment benefits, and alternative options available for treatment were discussed before obtaining the patients’ informed consent for the procedure. All patients were subjected to baseline laboratory investigations depending on their history and clinical findings. A coagulation profile, X-ray chest, and computed tomography were performed to exclude distant metastasis and pulmonary pathology. Angiography and Doppler ultrasound were performed pre-operatively to exclude any pre-existing pathological anomalies of the lower-leg vascular anatomy that may have caused impairments of circulation of the lower extremities. Patients requiring reconstruction of defects greater than 6 cm were selected for this study. Patients with systemic illness in whom it would have been problematic to execute surgery under general anesthesia, and those suspected of having deep-vein thrombosis were excluded from the study. Informed written consent was taken from all patients for inclusion in this study. The defects were classified according to Brown’s classification of mandibular defects, as shown in Table 1.

Moreover, the validated OHIP-14 [14,15] questionnaire translated into the Urdu language was used to assess the oral health-related quality of life of these patients 1 week before surgery, 1 month after surgery, and 1 month after rehabilitation.

### 2.3. OHIP-14

Oral Health Impact Profile scale comprised a total of 14 questions related to seven dimensions: functional limitation, physical pain, psychological discomfort, physical disability, psychological disability, social disability, and handicap. Each question in the OHIP-14 scale has 5 options, as follows: 0 = Never, 1 = Rarely, 2 = Sometimes, 3 = Repeatedly, and 4 = Always. In OHIP-14, each question has a numerical value that is added at the end to obtain a total score for each participant. On the Oral Health Impact Profile scale, the minimum score was 0, with 56 being the maximum, and a score above 10 indicated poor Quality of Life (QoL).

### 2.4. Tumor Resection Surgery and Flap Provision

**Figure 1:**
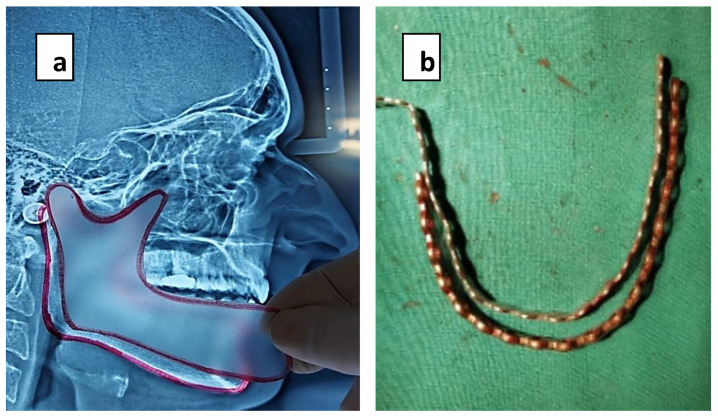
(**a**,**b**) Use of templates to shape the fibula into a new mandible.

Immediate repair and reconstruction after tumor resection were performed in all cases by two operating teams, one performing jaw resection and a second harvesting the flap. Before tumor resection and achievement of clear margins, a titanium reconstruction plate was temporarily fixed to the lower margin of the mandible in the location of anticipated mandibular segments. An aluminum template of the reconstruction plate was placed. It guided the reconstruction plate to be bent according to the mandibular curvature. A reconstruction plate was given to second team to shape the new mandible donor site before division of flap to minimize the ischemia time. The holes for the recon-plate and those of screws on the mandible were noted with markings to ensure that they were replaced in the exact position after resection and during flap in setting. The tumor was subsequently resected with safe margins, and the remaining mandibular segments were secured by mandibular maxillary fixation. A previously shaped transparent sheet of plastic was utilized to produce an outline and shape of the defect on lateral cephalogram (1:1). It was also used as a stencil to shape the harvested flap. Measurements were taken with help of a calibrated scale, which was also used for orientation of bony wedge to be removed between osteotomies (Figure 1a,b).

The second team was simultaneously involved in harvesting the flap. For pedicle orientation, whether anteriorly or posteriorly positioned, ipsilateral/contralateral limbs were selected as the donor tissue for fibula flap harvest. Evaluation and examination of the lateral limb were performed with a hand-held Doppler exact identification and the skin marking of the perforating vessels in the septum. For osteocutaneous flap, the skin flap was planned in such a fashion that would incorporate at least one vessel. The selected limb was raised for 5 min at 45° before tourniquet inflation using an Esmarch’s bandage for surgical hemostasis to facilitate draining of the blood vessels from the distal end to the proximal end, creating a clear surgical field with total blood loss reduction and decreasing post-tourniquet-release risk of microemboli formation. Tourniquet application was performed strictly according to Bruner’s ten rules for the safe use of tourniquet (modified by Braithwaite and Klenerman) [11].

**Figure 2:**
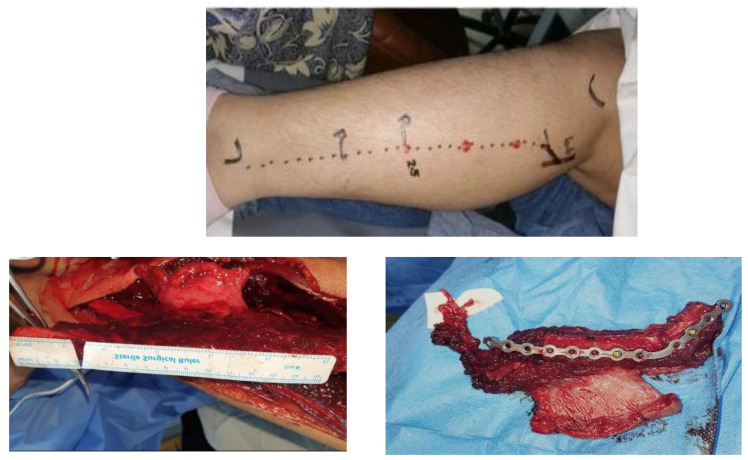
The landmarks and skin markings for fibular flap.

Subsequent to application of tourniquet, 2-step scrub, paint cleaning with Betadine Surgical Scrub (7.5% povidone-iodine), and proper draping, surgical incisions were completed over the markings placed on the skin beforehand. Following elevation and retraction of the skin flap, dissection was advanced in layers (steps: anterior retraction of fascia, peroneus longus, and final identification of the fibula bone). The identification and localization of the posterior crural septum were performed, and confirmation of perforators was made through visual examination. Transection of extensor halluces longus was performed. Dissection was extended posteriorly to release and separate the skin paddle from the soleus and gastrocnemius muscle. The thick interosseous septum was identified. Bone cuts were made with an oscillating saw with proximal preservation of 6 cm of bone and 8 cm of distal bone to avoid peroneal nerve injury and provide ample bone support for the ankle, respectively. The fibula was subsequently released for separation from surrounding tissues. and the flap pedicle was identified, dissected, and preserved, as shown in Figure 2.

The required length of fibula bone was finally measured with calibrated paper scale and retrieved after making multiple osteotomy cuts with the help of an oscillating saw for recontouring of the bone to give it the mandibular shape according to the prefabricated template and pre-plated reconstruction plates, before dividing the pedicle. The fibula transplant was plated with pre-plated recon-plate in the pre-marked screw holes and transferred and divided later into the mandibular defect once the recipient’s vessels were prepared for anastomosis. The pedicle was placed along the inner side of the flap and the lingual side of the mandible. The micro-vascular anastomosis of the flap was then performed in the recipient bed following standard techniques with facial and superior thyroid arteries, and external and internal jugular veins. After closure at the donor site, a plaster splint was fabricated for the donor limb and applied to the posterior aspects of the leg.

### 2.5. Statistical Analysis

The SPSS statistical version 25 was used for data analysis. By using simple descriptive statistics, the frequency and percentage were calculated for gender, site of tumor, defect type, type and size of flap, need for secondary flap for defect closure, type of closure at the donor site, postoperative course and recovery, flap take-up (full/partial/none), and complications at donor as well as reconstructed site. Numerical variables including age in years, operative time in hours, size of skin flap and size of defects in cm, and number of flaps required for reconstruction-site defect closure were analyzed using mean and standard deviations. The bivariate analysis through chi-square test was performed to detect any correlation between independent variables (gender, donor-site complication, recipient-site complication, and Brown’s classification (defect extent)) and the free vascularized fibular flaps used for reconstruction. A *p*-value ≤ 0.05 was considered significant.

## 3. Results

The study included a total of 18 patients with ages ranging from 24 to 63 and a mean age of 44.77 ± 12.05. Out of the total, 12 were male and 6 were female. There were 11 cases of squamous-cell carcinoma, 2 cases of ameloblastoma, and 1 case each of malignant melanoma, malignant nerve sheath tumor, giant-cell tumor, osteosarcoma, and chondrosarcoma. Furthermore, the tumors extended into the buccal vestibule in 12 cases, 2 cases involved the floor of the mouth, and 1 each case involved the lip, the palatal mucosa, the tongue, and the retromolar trigone region.

Additionally, in five cases, the defect measured approximately 17 cm. There were four cases with 10 cm, and four cases with defects of 16 cm, approximately. Another two cases had an 18 cm defect, while one case measured 15 cm. However, in 12 cases, the fibula flap was used as a single reconstruction option, and in 5 cases, two flaps (including the fibula and the supraclavicular flaps) were used in combination to reconstruct the defect. In one case, three flaps (including the fibula, the supraclavicular, and the pectoralis major) were utilized for reconstruction. Out of these cases, the fibula bone was double-barreled in two of the cases, while in the rest of the cases, the single-barrel technique was used. In four of the cases, the skin paddle measured approximately 6 × 12 cm, while in another four cases, it measured up to 7 × 14 cm. In three of the cases, the skin paddle used measured up to 6 × 10 cm, while in two cases, it was 4 × 7 cm in size. The rest of the defects required a skin paddle of 4 × 10 cm, 4 × 6 cm, 3.5 × 6 cm, and 3 × 5 cm for closure. Furthermore, the primary closure of the donor site was possible in 3 of the cases, while in the remaining 15 cases, split-thickness skin graft was used to complete closure.

In 12 cases, no complication occurred at the donor site. Three cases presented ecchymosis, while in two cases, there was a 1–2 cm wound dehiscence that later healed secondarily. In one of our cases, compartment syndrome also presented as a complication at the donor site, as described in (Table 2).

Moreover, at the recipient site, 15 cases presented no postoperative complications, while 2 cases had a partial failure of the extra-oral skin paddle, and 1 case had a complete failure of the extra-oral skin paddle. In 13 cases, there was full take-up of the flap, and in 4 of the cases, there was partial take-up. No take-up was observed in a single case, as shown in (Table 3).

**Figure 3:**
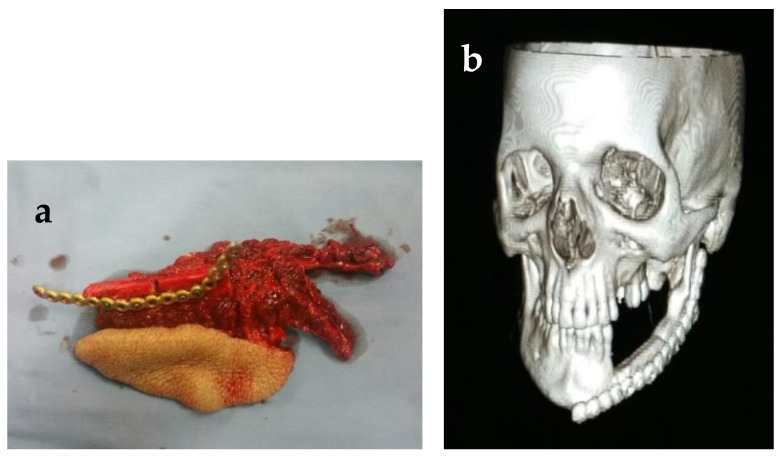
(**a**,**b**) Fibula graft, 3D computed tomography of patient showing hemimandibular defect reconstructed with fibula placed at the basal bone level.

**Figure 4:**
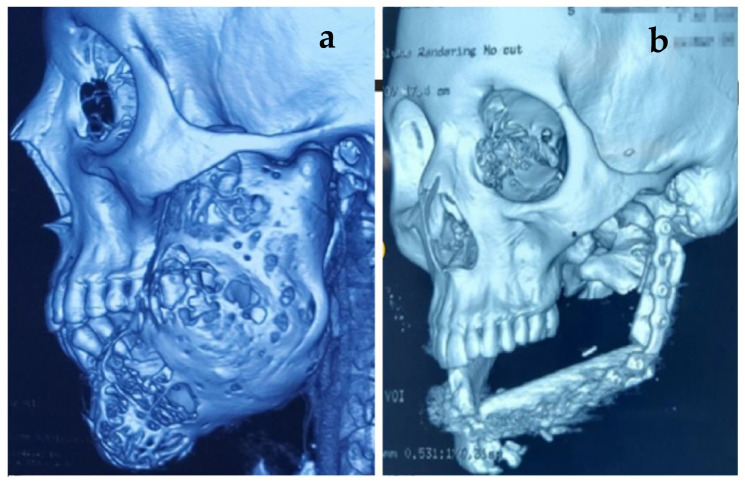
(**a**,**b**) 3D computed tomography of patient with ameloblastoma showing hemimandibular defect (Class II) reconstructed; transplanted fibula bone placed at the alveolar level to facilitate future implant rehabilitation.

**Figure 5:**
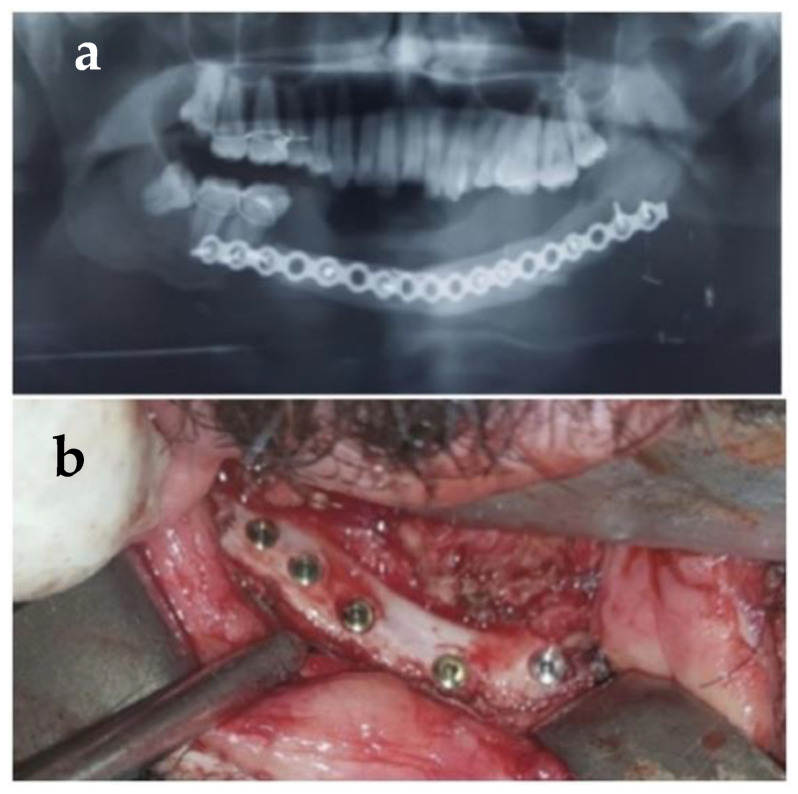
(**a**) OPG of patient showing reconstruction of Class III defect. (**b**) Class IV defect reconstruction in a patient with central giant-cell granuloma; transplanted fibula bone placed at the alveolar level.

The supraclavicular flap was used for the closure of skin defects in two cases, in one case as immediate, and in a second one to give extra-oral lining along with the fibula flap, which was utilized as a skin paddle for intra-oral lining. In cases where a loss of the skin paddle took place, the supra-clavicular flap was utilized to give coverage as extra-oral lining. Another case with a failed skin paddle was managed by using the pectoralis major as extra-oral lining. The follow-up 3D images were performed in subsequent visits to evaluate the form of the restored mandible, as shown in Figure 3, Figure 4 and Figure 5.

Moreover, there were nine angle-to-angle defects, three defects extending from the parasymphysis to the body, two cases extending from the body to the body of the mandible, two cases extending from the parasymphysis to the subcondylar region, and one maxillary defect, as shown in (Table 4).

Lastly, the distribution of the oral health impact profile scale is described in (Supplementary Table S1). The total OHIP-14 scores for patients before surgery, after surgery, and after dental rehabilitation were 12.03 ± 1.34, 10.66 ± 1.41, and 08.33 ± 0.62, respectively. The prevalence of different domains in the OHIP-14 scale before surgery was as follows: Functional limitation was seen in 11 participants when assessed by scores 3 and 4 of item Q1 and Q2. Physical pain was observed in 13 after summing up scores 3 and 4 of items Q3 and Q4. Psychological discomfort was found in five participants when evaluated through items Q5 and Q6. Physical disability was seen in four after estimation by items Q7 and Q8. Psychological disability was found in five when assessed by items Q9 and Q10. Social disability was evident in six participants when analyzed by items Q11 and Q12, and handicap was found in nine patients before surgery when investigated by items Q13 and Q14.

The prevalence of different domains in the OHIP-14 scale after surgery but before dental rehabilitation was as follows: Functional limitation was seen in 11 participants when assessed by scores 3 and 4 of items Q1 and Q2. Physical pain was observed in seven after summing up scores 3 and 4 of items Q3 and Q4. Psychological discomfort was found in eight when evaluated through items Q5 and Q6. Physical disability was seen in five after estimation by items Q7 and Q8. Psychological disability was found in seven when assessed by items Q9 and Q10. Social disability was evident in six participants when analyzed by items Q11 and Q12, and handicap was found in six participants when investigated by items Q13 and Q14.

The prevalence of different domains in the OHIP-14 scale after dental rehabilitation was as follows: Functional limitation was seen in three participants when assessed by scores 3 and 4 of item Q1 and Q2. Physical pain was observed in one after summing up scores 3 and 4 of items Q3 and Q4. Psychological discomfort was found in three when evaluated through items Q5 and Q6. Physical disability was seen in two after estimation by items Q7 and Q8. Psychological disability was found in one when assessed by items Q9 and Q10. Social disability was evident in one participant when analyzed by items Q11 and Q12, and handicap was found in three participants when investigated by items Q13 and Q14.

Additionally, the chi-square test analysis of free vascular flaps complication with independent variables (gender, donor- and recipient-site complication, and mandibular defects (Brown’s classification)) was performed. Gender showed a significant correlation (*p* = 0.030) when compared to the free vascular flaps used for reconstruction, with complications being more prevalent in males collectively (12 participants).

However, no significant difference was found when donor- recipient-, or mandibulectomy-site defects (Brown’s classification) were compared with free vascular flaps (*p* > 0.05), which shows that the complications reported were seen equally in both donor and recipient sites, as well as among all types of mandibulectomies (Brown’s classification), as presented in (Table 5).

## 4. Discussion

The reconstruction of defects with any composite flap is accompanied by several complications at both donor and recipient sites and by a range of medical complications [16,17]. Warraich et al. [2] reported an overall success rate of 82.6% in their sample of 17 cases reconstructed with fibula flap. They reported three complete graft failures, attributing one to an unfavorable recipient bed of firearm injury, the second to venous thrombosis, and the third to expiration because of blood transfusion complications. They reported vascular complications at the recipient site in two cases; six of their patients presented with wound infection, while three cases of wound dehiscence occurred. One of their patients developed skin and osseous graft necrosis. In comparison, no complication was observed at the donor site in 66.7% of our cases. However, three cases of ecchymosis and two cases of wound dehiscence (1–2 cm) that later healed secondarily were observed. In one patient, compartment syndrome presented as a complication at the donor site. Primary closure was performed, donor-site closure was released promptly, and anterior tibial vessels returned after some time. The donor-site skin defect was temporarily covered with bacteria’s dressing and later the skin was grafted. At the recipient site, 15 cases presented without any postoperative complications, while 2 cases had a partial failure of the extra-oral skin paddle, and 1 case had a complete failure of the extra-oral skin paddle. The overall success rate in our study was 72.2%, which is lower than in their study. All our patients survived the reconstructive procedure, and we did not observe any surgical site infection in any of our cases.

Similarly, Shah et al. [18] performed 56 free osteocutaneous flaps for mandible reconstruction and observed that, with the failure of only four flaps, their success rate was 91%. The complications reported by them included 13 cases of wound infections, skin graft at donor site being lost in 5 of their cases, 4 cases of complete flap loss, percutaneous fistulae in 3 cases, 2 cases of ankle instability, and one case each of skin-paddle necrosis and sensory deficit distal to the donor site. We did not observe any case of wound infection in our case series.

Furthermore, pre-operative planning and vascular imaging play a major role in the reduction in donor-site complications. We observed very few complications at the donor site, which can be attributed to the routine angiography and Doppler ultrasound performed pre-operatively to exclude any pre-existing pathological anomalies of the lower-leg vascular anatomy. Likewise, a cautious surgical dissection also significantly reduces donor-site morbidity [19].

Berrone et al. [20] reported six cases requiring reconstruction with a fibular osteo-cutaneous flap and recommended that the skin paddle should only be designed after the intraoperative identification of cutaneous branches under direct vision. Moreover, the skin-flap size should not surpass 6 × 10 cm in case a single perforator is incorporated to prevent the failure of the extra-oral skin paddle postoperatively [17]. We concur with their recommendation, as one of our flaps with dimensions over 6 × 10 cm presented with a complete failure of the extra-oral skin paddle.

Van Gemert et al. [16] performed 79 free fibula flaps to reconstruct the mandible from 1999 to 2013. They noted an increased risk of early complications needing surgical intervention while reconstructing across the midline defects of the mandible. Similarly, in 2016, Lodders et al. [21] described an increased risk of postoperative complications in continuity defects of the mandibular anterior region in reconstructions with fibular free flaps.

In addition, our findings were consistent with those of both Van Gemert et al. [16] and Lodders et al. [21], as three out of seventeen of our cases involving the anterior mandibular symphysial region presented with postoperative complications at the reconstruction recipient site. The association of an increased incidence of postoperative complications with the anterior mandibular symphysial region is attributable to multiple factors, including the anatomical complexity demanded by this area during the reconstruction procedure; the larger surgical wound created due to the bilateral neck dissection; the extensive surgical dissection to facilitate accessibility to the mandible mandated in such defects; and the resultant longer operation time of more than ten hours due to these factors [19]. The overall success rate in our study was 72.2%, which is lower than the 94.50% reported in a recent systematic review and network meta-analysis by Mashrah et al. [22]. This may be attributed to the fact that almost all of our cases included large segmental defects that extended across the midline of the mandible. In addition, other factors might have also played a role, such as the patients’ general condition, the surgeon’s experience, the perioperative fluid and medications, the length of the operation, etc.

Moreover, none of the previous studies have evaluated/compared the quality of life of these patients at all three stages, i.e., before surgery, after surgery, and after dental rehabilitation. In the present study, OHRQoL markedly improved after surgery and dental rehabilitation. The total OHIP-14 scores for patients before surgery, after surgery, and after dental rehabilitation were 12.03 ± 1.34, 10.66 ± 1.41 and 08.33 ± 0.62, respectively. Functional limitation improved after rehabilitation and surgery from 61.11% to 16.66%. Psychological discomfort, which was 27.77% initially, also increased after surgery to 44.44%, which could be due to an improvement in compromised esthetics and surgical complications. Additionally, physical disability in patients improved after dental rehabilitation from 22.12% to 11.23%, along with social disability going from 33.33% to 5.55%, and handicap, from 50.11% of patients to 16.66%. These results are comparable to other studies where the percentage of subjects experiencing moderate to severe limitations in food choices after the resection and reconstructive surgery but before oral rehabilitation increased from 61% to 78% [23,24]. Contrarily, two studies evaluated the quality of life and found no statistically significant changes after dental rehabilitation except for the social aspect [12,13]. The differences in the results could be due to the retrospective nature of these studies. In addition, comparing the functional outcomes of patients who received a VFFF for the reconstruction of a jaw defect is generally hampered by diversity in the bone and soft tissue defects of these patients. This study revealed a significant association (*p* = 0.030) of gender with free fibular flap complications. This difference in both sexes is of limited impact and may be due to the difference in the male-to-female ratio (12 to 6), not due to the presence of actual differences in terms of free vascular graft complications.

## 5. Limitations

The limitation of our study was the small sample size of only eighteen patients. Large mandibular defects that approach 6 cm in size and require surgical reconstruction are rare among the general population. Any pathological lesion involving the mandible causing a large swelling of the face and asymmetry is often noticed at an early stage before acquiring a size big enough to warrant resection and reconstruction with a free fibula flap.

## 6. Conclusions

The free fibula flap is a reliable method for the microvascular reconstruction of maxillofacial defects, as it provides an adequate bone length that is readily adaptable to the remaining mandible structure, and it constitutes adequate support for dental implants as well. The overall success rate of FFF in our study was 72.2%, which is lower than that reported in the literature. This may be attributed to the fact that almost all of our cases included large segmental defects that extended across the midline of the mandible. A higher percentage of success can be guaranteed essentially through a tremendously cautious and gentle surgical approach. The oral health-related quality of life was markedly improved after the reconstruction of mandibular defects with free vascularized fibular flaps and dental rehabilitation. Therefore, it remains the ‘gold standard’ rehabilitation approach. However, long-term studies with a larger population and minimal bias are needed.

## Figures and Tables

**Table 1:** Description of Brown’s classification.

Brown’s Classification	Description
Class I	(Angle)—Lateral defect not including ipsilateral canine or condyle
Class IC	(Angle and condyle)—Lateral defect including condyle
Class II	(Angle and canine)—Hemimandibulectomy including ipsilateral but not contralateral canine or condyle
Class IIC	(Angle, canine, and condyle)—Hemimandibulectomy including condyle
Class III	(Both canines)—Anterior mandibulectomy including both canines but neither angle
Class IV	(Both canines and at least one angle)—Extensive anterior mandibulectomy including both canines and one or both angles
Class IVC	(Both canines and at least one condyle)—Extensive anterior mandibulectomy including both canines and one or both condyles

C—condyle involvement in resection.

**Table 2:** Frequency of donor-site complications (*n* = 18).

Donor-Site Complication	Frequency
No complications	12
Ecchymosis	3
1 to 2 cm wound dehiscence, healed secondarily	2
Compartment syndrome	1

**Table 3:** Frequency of recipient-site complications (*n* = 18).

Recipient-Site Complication	Frequency
No complications	15
Partial failure of the extra-oral skin paddle	2
Failure of the extra-oral skin paddle	1

**Table 4:** Distribution of defect extent.

Brown’s Classification	Frequency
Class I defect	7
Class IV defect	1
Class II defect	4
Class III	3
Class IC	1
Class IIC	1

C—condyle.

**Table 5:** Association of independent variables with free vascular flaps (*n* = 18).

Variables		Gender (Frequency)		Donor-Site Complication (Frequency)	Recipient-Site Complication (Frequency)	Brown’s Classification (Frequency)
		Male	Female			
Free vascular fibula flaps	Single flap	2	0	5	2	0
	Two flaps	1	1	1	1	5
	Three flaps	1	1	0	0	2
No complication		8	4	12	15	11
Chi-square		2.400		2.125	1.090	8.316
df		2		6	4	10
Standard error		0.254		0.120	0.159	0.185
Spearman Correlation		0.055		0.029	0.034	0.218
*p*-value		0.030		0.908	0.896	0.518

Single flap: fibula; two flaps: fibula with supraclavicular flap; three flaps: fibula and supraclavicular with pectoralis major muscle; df—degree of freedom.

## Data Availability

The raw data used to support the findings of this study are included in the article.

## Supplementary Materials

The following supporting information can be downloaded at: https://www.mdpi.com/article/10.3390/healthcare11020193/s1, Table S1: Distribution of OHIP-14 scale responses (*n* =18).
