# Supplementary material for: Outcome Assessment after Reconstruction of Tumor-Related Mandibular Defects Using Free Vascularized Fibular Flap—A Clinical Study
Source: Healthcare (Basel). 2023 Jan 9;11(2):193. doi: 10.3390/healthcare11020193 (PMC9859578; doi:10.3390/healthcare11020193)
Supplement: Supplementary file 1 [file healthcare-11-00193-s001.zip › healthcare-2034208-supplementary.pdf]

Supplementary Table S1. Distribution of OHIP-14 scale responses (*n* =18).

Q	OHIP-14 scale	The stage when the questionnaire was filled	Never (N)	Rarely (N)	Sometimes (N)	Repeatedly (N)	Always (N)
1.	**Have you had trouble pronouncing any words?**	Before surgery	3	2	8	4	1
		After surgery	2	2	5	6	3
		After dental rehab	4	12	1	1	0
`2.	**Have you felt that your sense of taste has worsened?**	Before surgery	2	2	9	2	3
		After surgery	1	1	13	2	1
		After dental rehab	11	3	3	1	0
3.	**Have you had painful aching in your mouth?**	Before surgery	0	1	2	12	3
		After surgery	8	7	3	0	0
		After dental rehab	16	1	1	0	0
4.	**Have you found it uncomfortable to eat any foods?**	Before surgery	1	1	9	4	3
		After surgery	0	2	9	4	3
		After dental rehab	16	1	1	0	0
5.	**Have you been self-conscious?**	Before surgery	0	2	7	9	0
		After surgery	1	3	13	1	0
		After dental rehab	16	2	0	0	0
6.	**Have you felt tense?**	Before surgery	0	1	16	1	0
		After surgery	0	2	16	0	0
		After dental rehab	13	2	3	0	0
7.	**Has your diet been unsatisfactory?**	Before surgery	8	4	3	2	1
		After surgery	12	1	5	0	0
		After dental rehab	9	7	1	1	0
8.	**Have you had to interrupt meals?**	Before surgery	0	1	8	8	1
		After surgery	0	6	8	4	0
		After dental rehab	9	9	0	0	0
9.	**Have you found it difficult to relax?**	Before surgery	0	1	7	8	2
		After surgery	0	2	9	6	1
		After dental rehab	15	2	1	0	0
10.	**Have you been a bit embarrassed?**	Before surgery	0	2	9	5	2
		After surgery	3	4	5	3	3
		After dental rehab	17	1	0	0	0
11.	**Have you been a bit irritable with others?**	Before surgery	0	0	16	2	0
		After surgery	2	5	9	2	0
		After dental rehab	9	8	1	0	0
12.	**Have you had difficulty doing your usual job?**	Before surgery	0	3	10	4	1
		After surgery	0	0	15	2	1
		After dental rehab	17	1	0	0	0
13.	**Have you felt that life, in general,**	Before surgery	0	0	3	6	9
		After surgery	0	2	9	4	3

	**was less satisfying?**	After dental rehab	9	7	2	0	0
14.	**Have been unable to function?**	Before surgery	0	0	10	6	2
		After surgery	0	1	6	9	2
		After dental rehab	2	15	1	0	0

N: frequency
